# Editorial: Microbiota, nutrition and stress: modulators of immunity

**DOI:** 10.3389/fnut.2023.1328346

**Published:** 2023-11-17

**Authors:** Sergey Ponomarev, Isabelle Mack, Alexander Choukér

**Affiliations:** ^1^Laboratory of Immune System Physiology, State Scientific Center of Russian Federation, Institute of the Biomedical Problems, Russian Academy of Sciences, Russian Federation, Moscow, Russia; ^2^Department of Psychosomatic Medicine and Psychotherapy, University Hospital Tübingen, Tübingen, Germany; ^3^Laboratory of Translational Research ‘Stress and Immunity', Department of Anesthesiology, LMU University Hospital, Ludwig-Maximilians-Universitó Munich (LMU), Munich, Germany

**Keywords:** adaptive immunity, innate immunity, microbota, nutrition, stress

Vertebrate immunity is a complex system that involves a large number of cells of different organs and intracellular pathways in charge of the protection and maintenance of the body's integrity and homeostasis. Inefficient coordinated action and balancing functions of the complex interplay of the human immune system increase the risk for acute and deadly infectious diseases, while excessive immune responses may lead to autoimmune disorders and subsequent morbidity. Unlike other body systems, immunity functioning patterns are highly individual and genetics only partially defines them. Along with the host immune system, the microbiota of the host is thought to be pivotal in the maintenance of human health by affecting energy metabolism, intestinal function, and immune response among others. In addition, environmental factors such as diet and stress exposures on the one hand, and intrinsic factors such as age and sex on the other hand, modulate immunity by altering gene expression patterns within immunity-related cells, shifting their number in subpopulations and affecting their phenotype and key properties. In the 10 articles within the current Research Topic, these aspects are considered, creating an opportunity to have an idea of the factors that determine the functioning of immunity in animals and humans, respectively.

“It is the start of a new era when people are finally ready to embrace the microbial world.” This anticipated direction has been truly voiced by the science journalist and Pulitzer prize winner Yong (1), and the microbial role in the concept of the intestine-immunity axis is widely accepted today (2). Both animal and human studies confirm the potential role of gut microbiota in immunomodulation (3). Interestingly, Hou et al. revealed the causal relationships between gut bacteria and gout, a widespread metabolic disorder, which has a strong autoimmune potential (4). On the other hand, gut microbiota largely depends on a diet. Ali et al. report that food rich in dietary fibers positively affects the microbiome composition in meat geese, which, in turn, induces antimicrobial, antioxidant, and anti-inflammatory properties of gut barriers. At the same time, according to Mardi et al., a proper diet in humans enhances patient survival and improves the overall effect of COVID-19 therapy. Besides dietary fibers and pre- and probiotics, a healthy diet should include nutrients, namely, essential amino acids, choline, non-saturated fatty acids, carbohydrates, and vitamins. A diet rich in fat, as well as prolonged fasting, may result in ketoacidosis, which negatively affects immune system functioning. The mechanism of this impairment was recently revealed in the work of He et al., demonstrating that one of the ketone bodies, namely, β-hydroxybutyrate, enhances neutrophil adhesion by inhibiting autophagy. In the review of Khan et al., the anti-inflammatory and anti-oxidative role of methionine, lysine, and choline is discussed in relation to periparturient ruminants, while Zhang et al. established the antioxidant capacity of tryptophan in fish. Surprisingly, in an experimental setting in mice, the regular consumption of an acidic polysaccharide extracted from green parts of *Chuanminshen violaceum*, a Chinese endemic plant, significantly improved their state of health. Zou et al. managed to show lowered inflammatory and oxidation indices in the murine blood. Apparently, like dietary fibers, carbohydrates primarily benefit healthy gut bacteria, which mediates the positive effects of CVP-AP-I.

Enzymes' catalytic properties rely on cofactors, many of which are either vitamins or metal ions. Munteanu and Schwartz systemically review the role of vitamins and micronutrients for proper immunity functioning, discussing in detail the to-date data on the role of vitamins A, C, and D, as well as cholesterol in the development of cancer and their therapeutic usage. In addition to their role as transcription regulators and coenzymes, the fat-soluble vitamins (A and E), as well as vitamin C, are known to be a part of the non-enzymatic module of the antioxidant system in the human body, reducing inflammation processes as induced by ROS. Hung et al. propose intravenous vitamin C as a monotherapy to reduce mortality in critically ill patients although the underlying mechanism and optimal dose, inclusion, and exclusion criteria remain to be thoroughly elucidated, especially while other antioxidant therapy mono-approaches, e.g., using essential trace elements [e.g., selenium (Se)], have not shown the expected clinical effectiveness in critical care patients (5).

Stress factors do affect and hereby modulate the immune response effectiveness as a function of the intensity of the stressors. In the current Research Topic, there is only one paper by Klos et al. that links the effects of isolation or confinement as an environmental stress factor and human immunity. The authors systematically review and summarize the data on microbiota composition shifts in different human trials in isolated and confined environments. The results of the study indicate that internal factors that shaped the microbiota over time appeared to be the primary drivers of its composition and function in response to isolation in antigen-deprived conditions. However, a preliminary trend indicates a decreased diversity of gut microorganisms in people inhabiting extreme conditions, which raises concerns about the risk of dysbiosis and associated diseases, including immunity dysregulation (3). Additionally, besides microbiota, there is evidence that emotional stress experienced by people in confinement directly affects their immune system's effectiveness. Numerous experiments in which they model space flight factors, including short-term and prolonged isolation, demonstrate impaired immunity functioning (6). In this regard, potential immunologic countermeasures are being developed to minimize the risks of immune system dysfunction during space exploration full of different stress factors (7).

In summary, the results of the abovementioned studies and the data from the reviews give insight into complex factors affecting immunity functioning. The studies illustrate well the interdependence of nutrition, microbiota, and immunity. On the one hand, the immune homeostasis seems too prone to alterations due to its susceptibility to various environmental factors. On the other hand, one may consider the power of little efforts in the maintenance of a healthy lifestyle to keep immunity in balance.

## Author contributions

SP: Conceptualization, Writing—original draft. IM: Conceptualization, Supervision, Writing—review & editing. AC: Conceptualization, Formal analysis, Project administration, Writing—review & editing.

## References

[1] Yong E. I Contain Multitudes: The Microbes Within Us and a Grander View of Life. New York, NY: Random House (2016), p. 355.

[2] Jacobson A Yang D Vella M Chiu IM. The intestinal neuro-immune axis: crosstalk between neurons, immune cells, and microbes. Mucosal Immunol. (2021) 14:555–65. 10.1038/s41385-020-00368-1 33542493 PMC8075967

[3] Li X Hu S Yin J Peng X King L Li L. Effect of synbiotic supplementation on immune parameters and gut microbiota in healthy adults: a double-blind randomized controlled trial. Gut Microbes. (2023) 15:2247025. 10.1080/19490976.2023.2247025 37614109 PMC10453972

[4] Cabău G Crişan TO Klück V Popp RA Joosten LA. Urate-induced immune programming: consequences for gouty arthritis and hyperuricemia. Immunol Rev. (2020) 294:92–105. 10.1111/imr.12833 31853991 PMC7065123

[5] Bloos F Trips E Nierhaus A Briegel J Heyland DK Jaschinski U. Effect of sodium selenite administration and procalcitonin-guided therapy on mortality in patients with severe sepsis or septic shock: a randomized clinical trial. JAMA Int. Med. (2016) 176:1266–76. 10.1001/jamainternmed.2016.2514 27428731

[6] Ponomarev S Kalinin S Sadova A Rykova M Orlova K Crucian B. Immunological Aspects of Isolation and Confinement. Front Immunol. (2021) 12:697435. 10.3389/fimmu.2021.697435 34248999 PMC8264770

[7] Makedonas G Mehta S Choukèr A Simpson RJ Marshall G Orange JS. Specific immunologic countermeasure protocol for deep-space exploration missions. Front Immunol. (2019) 10:2407. 10.3389/fimmu.2019.02407 31681296 PMC6797618

